# Systemic Alterations of Cancer Cells and Their Boost by Polyploidization: Unicellular Attractor (UCA) Model

**DOI:** 10.3390/ijms24076196

**Published:** 2023-03-24

**Authors:** Alexander E. Vinogradov, Olga V. Anatskaya

**Affiliations:** Institute of Cytology, Russian Academy of Sciences, 194064 St. Petersburg, Russia

**Keywords:** oncogenesis, cancer, atavistic reversal, interactome, gene expression, phylostratigraphy, polyploidy

## Abstract

Using meta-analyses, we introduce a unicellular attractor (UCA) model integrating essential features of the ‘atavistic reversal’, ‘cancer attractor’, ‘somatic mutation’, ‘genome chaos’, and ‘tissue organization field’ theories. The ‘atavistic reversal’ theory is taken as a keystone. We propose a possible mechanism of this reversal, its refinement called ‘gradual atavism’, and evidence for the ‘serial atavism’ model. We showed the gradual core-to-periphery evolutionary growth of the human interactome resulting in the higher protein interaction density and global interactome centrality in the UC center. In addition, we revealed that UC genes are more actively expressed even in normal cells. The modeling of random walk along protein interaction trajectories demonstrated that random alterations in cellular networks, caused by genetic and epigenetic changes, can result in a further gradual activation of the UC center. These changes can be induced and accelerated by cellular stress that additionally activates UC genes (especially during cell proliferation), because the genes involved in cellular stress response and cell cycle are mostly of UC origin. The functional enrichment analysis showed that cancer cells demonstrate the hyperactivation of energetics and the suppression of multicellular genes involved in communication with the extracellular environment (especially immune surveillance). Collectively, these events can unleash selfish cell behavior aimed at survival at all means. All these changes are boosted by polyploidization. The UCA model may facilitate an understanding of oncogenesis and promote the development of therapeutic strategies.

## 1. Introduction

The ‘War on Cancer’ was declared in 1971. Now, after a half hundred years, this war is not yet won, albeit certain progress in patients’ survival taking place, mostly because of medical programs of early detection [1]. Possibly, this unsatisfactory state of the art is due to a lack of clear understanding of the nature of cancer and its origin. The phenomenology is well outlined in the hallmarks of cancer first formulated by Hanahan and Weinberg and extended later [2,3,4,5,6]. The main hallmarks include proliferative advantage, replicative immortality, inducing/accessing vasculature, invasion and metastasis, reprogramming cellular metabolism, avoiding immune destruction, dedifferentiation, and transdifferentiation [2,3,4,5,6].

However, albeit the hallmarks providing a fine description of what goes wrong in cancer cells, they do not explain why those alterations appeared [4]. The prevailing paradigm—a classic gene-centered ‘somatic mutation’ theory (SMT)—suggests that cancer is caused by an alteration in a limited number of special genes (in simple formulation, gain of function in oncogenes or loss of function in tumor suppressors) [7,8]. However, recent discoveries raised issues in the SMT. Even the former active proponents of SMT raised concerns recognizing an increasing complexity of the problem [7]. The main issues with the SMT are as follows. 

The gene mutation pattern shows remarkable intratumor differences and significant changes over time, from the early tumor development up to the spreading of distant metastases [8]. A distinct set of genes shows mutations during different stages of cancer development, and doubts about the causative role of these genes are mounting [8]. The expression array analyses of various breast cancers showed that different gene sets were equally useful in predicting future clinical behavior but contained almost no genes in common [9,10]. Several important mutations were found only in a limited percentage of cancer cells of the same tumor, whereas a significant part of neoplastic cells do not show these mutations [11,12]. In a similar vein, mutations usually associated with cancer were also found in noncancerous tissues [13,14,15]. Importantly, the mutation load and spectra of normal cells resemble those of cancer cells [16]. As for the latter, a bibliometric study showed that nearly every gene (87.7%) was considered in the literature in association with cancer [17]. The multitude of altered molecular mechanisms were identified in cancer cells, most with low clinical predictability [18].

Furthermore, there is a discrepancy between the experimental data obtained on rodent models and human cells. Cancer transformation caused by the overexpression of oncogenes was achieved mostly in rodents, whereas human cells display a remarkably high resilience to these manipulations [19,20,21]. Mutated oncogenes, when incorporated into normal human cells, failed to immortalize or transform them [19]. The rodent cells are much more easily immortalized, which is one of the hallmarks of cancer [21]. This is probably because of a special way of evolution in the murine-like rodents, where natural selection favored the speed of development and reproduction at the expense of reliability of organization [22,23]. These results suggest an involvement of systemic factors, which cause the discrepancy between the human and rodent data.

Most carcinogens are mutagenic, yet up to 20% of known carcinogens lack genotoxic activity [24,25]. Furthermore, for different chemicals, the correlation between mutagenic and carcinogenic potencies is rather weak (r~0.3) [24]. The role of non-genetic plasticity in the origin and development of cancer is increasingly emphasized [26,27,28,29]. Generally, there is a paradox: depending on context, both the increase and decrease in a certain molecular player can correlate positively with tumor malignancy [8]. There is an opinion that the reductionist framework (gene-centered approach) can be inadequate for grasping the complexity of oncogenesis [8,18,29,30]. 

These issues evoked interest in the more systemic concepts dealing with the context of gene activity, which can be cell- or tissue-centered. The most important for our study is the cell-centered atavistic theory, suggesting that cancer is an evolutionary reversal to a unicellular state [31,32,33,34]. The genes of unicellular (UC) origin are overexpressed in cancer tissues, whereas the genes appearing at multicellular (MC) evolutionary stages are downregulated [35,36,37]. The human gene coexpression network and protein interaction network contain the giant cluster strongly enriched in the genes of UC origin and corresponding functions [38,39]. The expression of this cluster is upregulated in cancers [38,40]. A recent refinement of the atavistic theory (‘serial atavism’) suggests that cancer onset and progression involve a series of reversals [34]. Notably, the atavistic model relates rather to adult than childhood cancers, which are quite different and much less frequent than cancers associated with aging [34].

The other cell-centered, ‘cancer attractor’ theory suggests that oncogenesis is caused by a shift in cellular program activity towards a cancer attractor (stable state of cellular systems), which is achieved mostly via epigenetic mechanisms [29,41,42]. However, with a minor exception of transmissible tumors, cancers are not inheritable and reduce the fitness of a host organism. Therefore, it is unlikely that natural selection could create a special cancer attractor. Yet, a hybridization of this concept with the atavistic theory can solve this problem. The clusters of protein interaction networks have denser interactions within than between them, therefore they may serve as attractors for cellular programs. Importantly, the UC giant cluster shows a higher inside/outside connection ratio compared with the MC clusters, which suggests a stronger attractor effect [39]. In other words, evolutionary history can explain the appearance of the UC attractor (arising because of activation of ancient UC programs) but not of a particular cancer attractor.

The cell-centered ‘genome chaos’ theory assumes that oncogenesis is caused not so much by point mutations but rather by a large-scale genome rearrangement [18,30]. Chromosome instability is a typical feature of cancer cells [43,44]. A special assumption of the ‘genome chaos’ theory is that this genome rearrangement can be adaptive under stressful conditions [18,30]. In other words, chromosome recombination allows cancer cells to search for survival via genetic and epigenetic alterations (genes in the altered genome environment can change their expression pattern). This theory can be reconciled with the ‘atavistic reversal’ because it also assumes the appearance of selfish UC-like behavior of cancer cells, which was acquired during the evolutionary history. 

The tissue-centered ‘tissue organization field’ theory (TOFT) suggests that gene mutations are not the cause of oncogenesis but rather its by-products [8,45,46]. According to the TOFT, cancer is caused by the disruption of normal tissue organization. The cases of tumor suppression by the replacement in the normal microenvironment support this theory [47]. Another supporting fact is the foreign-body-based carcinogenesis, when tumors develop in the proximity of inserted materials, devoid of chemical activity [48,49]. The insertion of a foreign body can cause local tissue disorganization that is followed by oncogenesis. The metastases, when cancer cells proliferate in the novel microenvironment, seemingly contradict the TOFT. However, only a very tiny part (<0.02%) of invading cells develop macro-metastases [50], which suggests that most cancer invasions are consistent with the TOFT. The TOFT assumes that the default internal cell state in MC organisms is proliferation as in UC organisms, which is bounded only by tissue constraints [8,45,46]. This assumption is an overstatement because cells in MC organisms are very different. If placed in the culture (i.e., when there are no tissue constraints), the terminally differentiated cells do not proliferate at all, while the others proliferate only in the presence of growth factors [51,52]. Were proliferation the default state of normal cells, the growth factors were not needed, and even with growth factors, proliferation of cells from adult MC organisms is constrained by the Hayflick limit [53,54]. However, after replacement of ‘default proliferation’ by a UC attractor, the TOFT can be reconciled with the ‘atavistic reversal’ theory.

On the grounds of bioinformatic analyses performed here, we propose a unicellular attractor (UCA) model, which integrates essential features of the ‘atavistic reversal’, ‘cancer attractor’, SMT, ‘genome chaos’, and ‘tissue organization field’ theories. We propose a possible mechanism of the ‘atavistic reversal’ and its refinement called ‘gradual atavism’. In addition, we provide evidence supporting the ‘serial atavism’ model, which is a recent refinement of the ‘atavistic reversal’ theory [34].

## 2. Results

### 2.1. The Unicellular Attractor (UCA) in the Human Interactome

**Figure 1:**
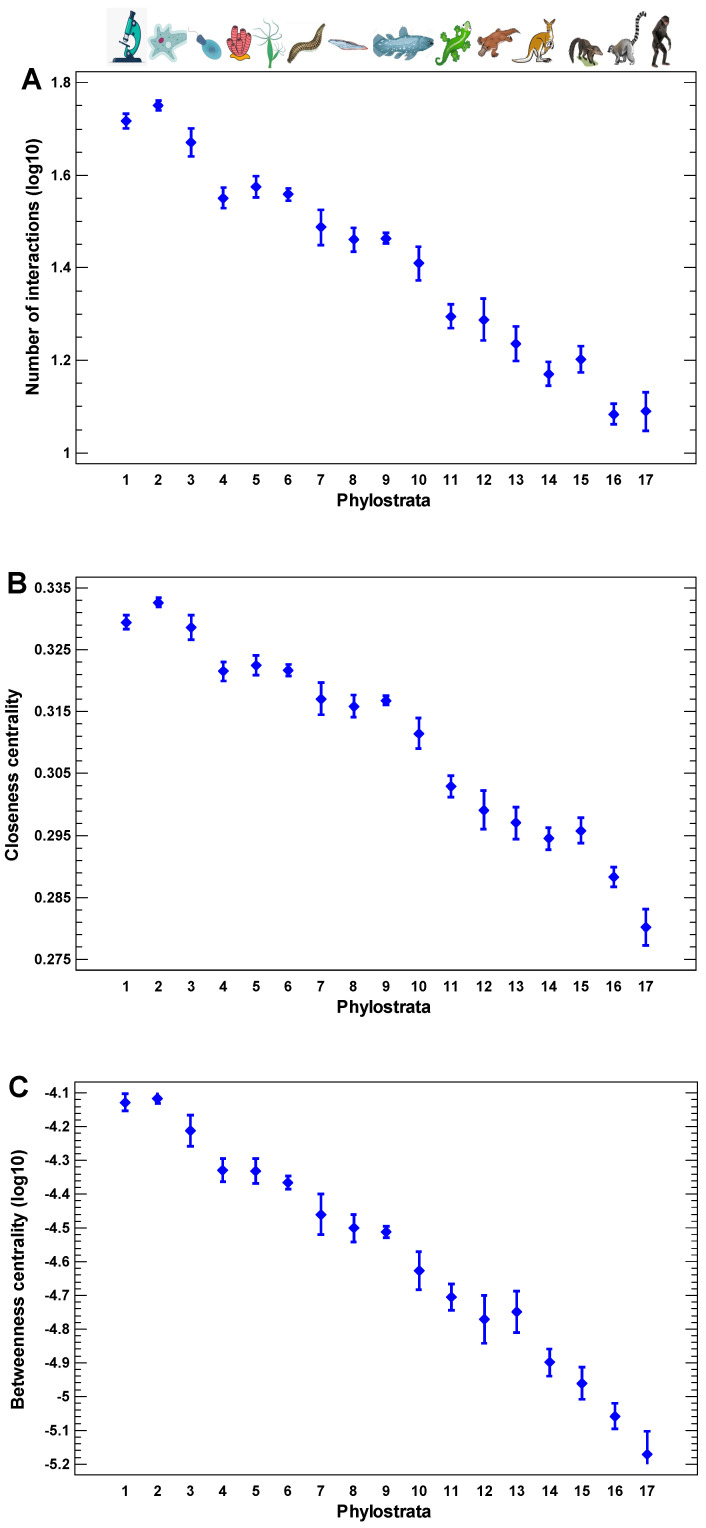
The evolutionary profile of local interaction density and global centrality measures in the human interactome. (**A**) The number of direct (one-step) interactions (degree). (**B**) Closeness (the reciprocal of the sum of the length of the shortest paths between a protein and all other proteins). The more central a protein is, the closer it is to all other proteins in the interactome. (**C**) Betweenness (the number of the shortest paths between all pairs of other proteins passing through a given protein). The more central a protein is in the network, the higher the number of the shortest paths passing through it. The protein interactions are from the STRING database [55], and the gene phylostratic mapping (shallow) is from [56]. Phylostrata: 1—cellular organisms (Prokaryota); 2—Eukaryota; 3—Opisthokonta; 4—Metazoa; 5—Eumetazoa; 6—Bilateria; 7—Chordata; 8—Vertebrata; 9—Euteleostomi; 10—Tetrapoda; 11—Amniota; 12—Mammalia; 13—Theria; 14—Eutheria; 15—Boreoeutheria; 16—Primates; 17—Hominidae. The pictures at the top show recent organisms corresponding to phyletic branching used for human gene dating.

The human protein interaction network demonstrates the gradual core-to-periphery evolutionary growth (Figure 1A–C). Both the local and the global centralities of the human interactome decrease with the decrease in the evolutionary age of encoding genes. The local centrality is defined by the number of direct (one-step) interactions of a protein (Figure 1A). The global centralities are presented by two measures: closeness and betweenness (Figure 1B,C). The closeness is the reciprocal of the sum of the length of the shortest paths between a protein and all other proteins. The more central a protein is in the network, the closer it is to all other proteins. The betweenness is the number of the shortest paths between all pairs of other proteins passing through a given protein. The more central a protein is in the network, the higher the number of the shortest paths passing through it. These observations show that the products of younger genes tend to participate on the periphery of the interactome. Therefore, the ancient network center may serve as an attractor for cellular programs in the cases of random alterations in protein interactions.

This suggestion was tested by the modeling of random walks along the protein interaction trajectories in the human interactome. A walk started from one of the youngest proteins (belonging to the 17th phylostratum), taken randomly. This was the first protein. From all its interactants, one was chosen randomly (second protein), and the next step started already from this protein, again to a randomly chosen next interactant (third protein), and so on. The reverses to the first and other previous proteins were allowed. The series of walks of a different length (from 5 to 10,000 steps) were tested. To ensure statistical significance, there were 10,000 repeats of each random walk, each repeat starting randomly from one of the proteins in 17th phylostratum. The number of repeats, which ended in each phylostratum, was normalized to the number of genes belonging to this phylostratum. We assume that these random walks can simulate random alterations in the interactome caused by mutations in encoding genes or disturbances in gene expression or protein configuration. It was shown previously that alterations in protein interactions are associated with changes in gene expression [57].

**Figure 2:**
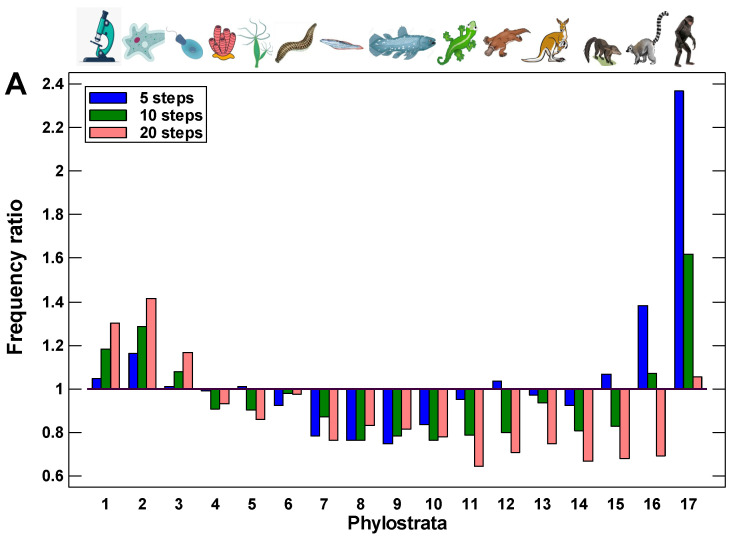

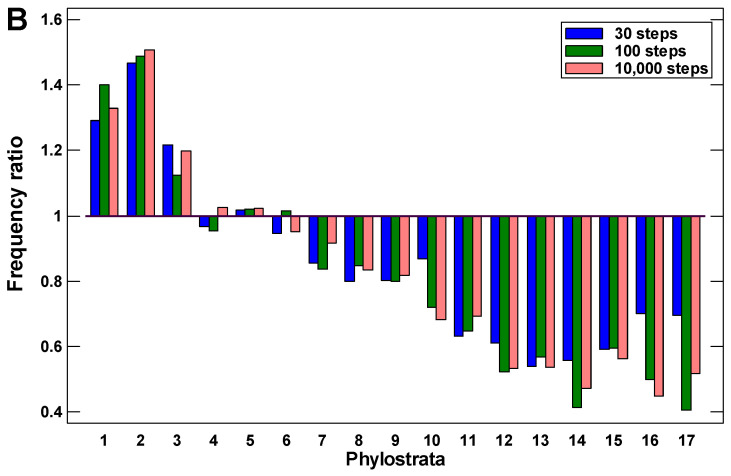
The results of random walks along the protein interaction trajectories in the human interactome: the frequency of walk ends in each phylostratum normalized to gene number in this phylostratum (all walks began in 17th phylostratum): (**A**) walks of 5–20 steps long; (**B**) walks of 30–10,000 steps long. The enrichment of walk ends in the UC center is highly significant (beginning from 10 steps, *p* < 10^–16^). Phylostrata: 1—cellular organisms (Prokaryota); 2—Eukaryota; 3—Opisthokonta; 4—Metazoa; 5—Eumetazoa; 6—Bilateria; 7—Chordata; 8—Vertebrata; 9—Euteleostomi; 10—Tetrapoda; 11—Amniota; 12—Mammalia; 13—Theria; 14—Eutheria; 15—Boreoeutheria; 16—Primates; 17—Hominidae. The pictures at the top show recent organisms corresponding to phyletic branching used for human gene dating.

The random walks ended much more frequently in the UC phylostrata than in the MC phylostrata (Figure 2). Already after a few steps, the walk ends begin to appear in the UC phylostrata, with a depression between the most recent phylostratum (from which the walks started) and the UC center (Figure 2A). This observation indicates the existence of the UC attractor. With the increase in the number of steps, the frequency of walks ending in the UC phylostrata only grows. After about 30 random steps, the frequency of walks ending in the UC phylostrata stabilizes, indicating a balanced state (Figure 2B). 

There are three phases in the stabilized picture: (i) the peak in the 1–3 phylostrata (UC) with a frequency that is higher than expected from the number of genes belonging to these phylostrata, (ii) the plateau in the 4–6 phylostrata (Metazoa-Bilateria) with a frequency that is similar to expected, and (iii) the lower than expected frequency in the latter phylostrata gradually declining towards more recent times (Figure 2B). These results suggest that random alterations in protein interactions can cause gravitation of protein interaction activity towards the UC center, which indicates the existence of the UC attractor. The strength of this attractor gradually declines towards younger proteins. 

### 2.2. Gene Expression Levels in Different Phylostrata

**Figure 3:**
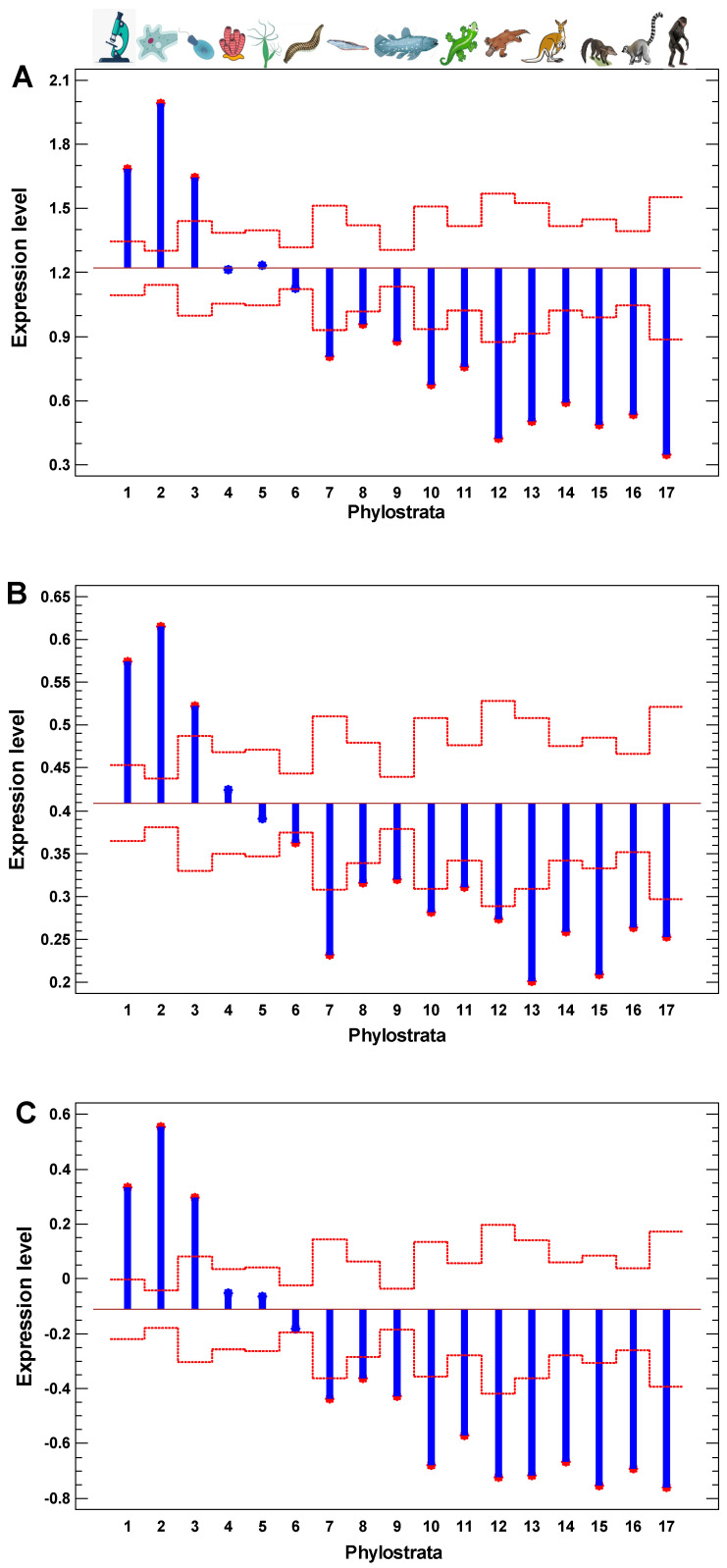
The evolutionary profile of gene expression levels in normal and non-invasive cancer cells. The analysis of means (ANOM) plot showing which phylostrata means are different from the total mean: (**A**) normal cells in the ‘breast cancer’ dataset; (**B**) normal cells in the ‘melanoma’ dataset; (**C**) non-invasive cancer cells in the ‘myeloma’ dataset. Red dotted lines show confidence intervals for individual phylostrata (*p* = 0.05). Phylostrata: 1—cellular organisms (Prokaryota); 2—Eukaryota; 3—Opisthokonta; 4—Metazoa; 5—Eumetazoa; 6—Bilateria; 7—Chordata; 8—Vertebrata; 9—Euteleostomi; 10—Tetrapoda; 11—Amniota; 12—Mammalia; 13—Theria; 14—Eutheria; 15—Boreoeutheria; 16—Primates; 17—Hominidae. The pictures at the top show recent organisms corresponding to phyletic branching used for human gene dating.

Similar to the interactome centrality measures and the results of random walk modeling along protein interaction trajectories, the level of gene expression is also higher in the UC genes and declines towards younger genes (Figure 3A). Roughly, there are the same three phases as in Figure 2B: (i) the peak in the 1–3 phylostrata (UC), (ii) the plateau in the 4–6 phylostrata (Metazoa-Bilateria), and (iii) the decline in the latter phylostrata. This observation indicates that the UC center of cellular networks is maintained in the more active state, compared with more recent periphery. Notably, there is a minor distortion in the monotonity of decline across the UC phylostrata in all tested measures—interactome centrality, random walk modeling, and gene expression levels. The genes from the second phylostratum show higher values than the genes from the first phylostratum. This exception suggests that after the origin of eukaryotes (at the second phylostratum), the informational processes dealing with epigenetic regulation (chromatin maintenance and modification) put forward on the central place in cellular networks (interactome and transcriptome) at the expense of metabolic pathways, which appeared mostly at the first phylostratum. 

### 2.3. Expression of Ancient Genes Is Upregulated in Cancer Cells in a Gradual Way

**Figure 4:**
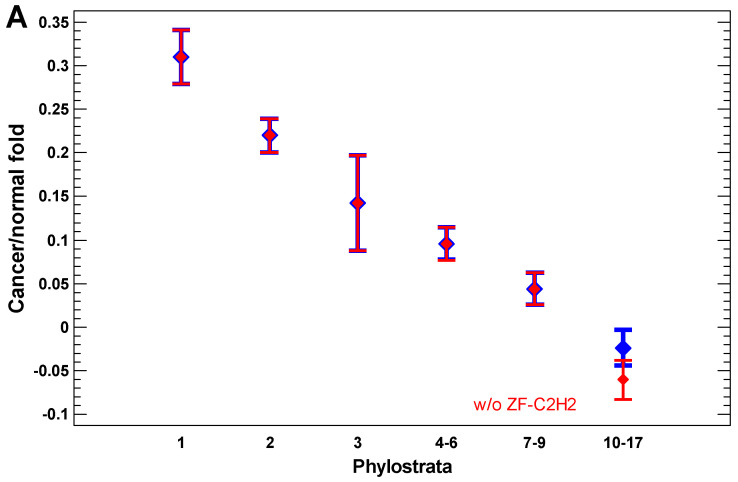

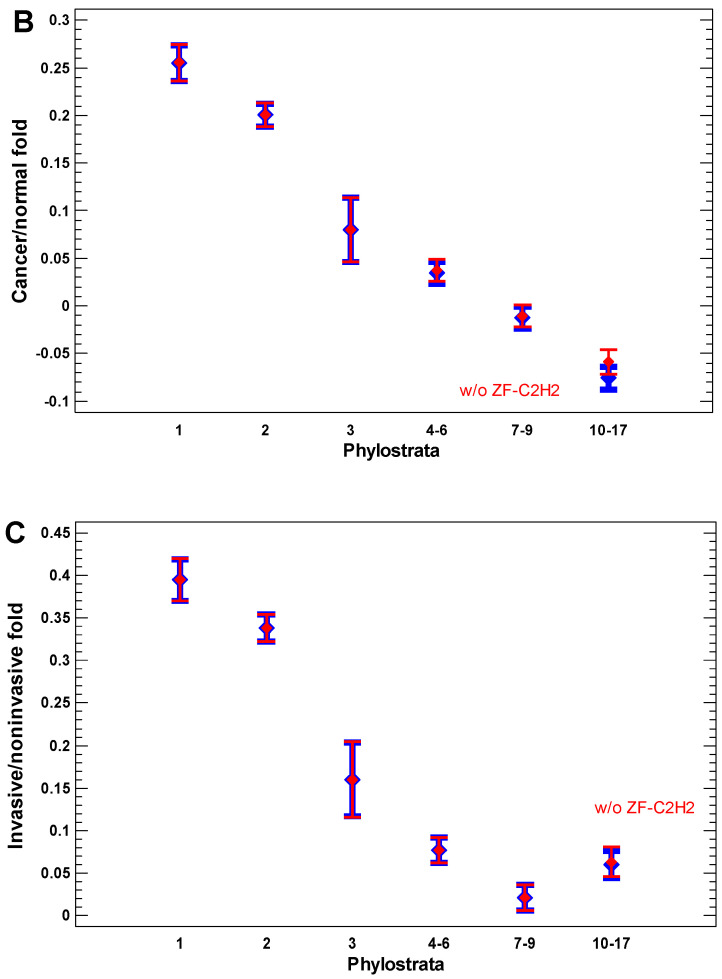
The evolutionary profile of gene expression folds: (**A**) cancer/normal fold in the ‘breast cancer’ dataset; (**B**) cancer/normal fold in the ‘melanoma’ dataset; (**C**) invasive/non-invasive fold in the ‘myeloma’ dataset. Because the detailed picture for all 17 phylostrata is noisy, the folds for consecutive phylostrata were averaged, roughly in accordance with the expression levels in Figure 3. The data without ZF-C2H2 genes are shown in red. The moderate activation of ZF-C2H2 genes in breast cancer cells can be seen (**A**). Phylostrata: 1—cellular organisms (Prokaryota); 2—Eukaryota; 3—Opisthokonta; 4—Metazoa; 5—Eumetazoa; 6—Bilateria; 7—Chordata; 8—Vertebrata; 9—Euteleostomi; 10—Tetrapoda; 11—Amniota; 12—Mammalia; 13—Theria; 14—Eutheria; 15—Boreoeutheria; 16—Primates; 17—Hominidae.

In the cancer cells, the expression of older genes is enhanced further, as can be seen from the cancer/normal fold of expression level (Figure 4A,B). In the invasive cancer cells, it is enhanced even more as compared with the non-invasive cancer cells (Figure 4C). The gradual increase in the cancer/normal fold towards more ancient phylostrata is in accordance with the interactome centrality measures and random walk modeling, which showed a similar gradual shift towards the UC phylostrata. This phenomenon can be called the ‘gradual atavism’. The similar increase in the invasive/non-invasive cancer fold (Figure 4C) supports the ‘serial atavism’ model, which states that cancer onset and progression involve a series of atavistic reversals [34]. Notably, in the case of expression folds, there is no distortion of the ‘gradual atavism’ among the 1–2 phylostrata, which was seen in the interactome centrality, random walk modeling, and expression levels. Probably, metabolism (involving predominantly genes from the first phylostratum) becomes relatively more important for cancer cells than epigenetic regulation (involving genes from the second phylostratum). 

The zinc finger C2H2 transcription factors (TF ZF-C2H2), which are expanded via gene duplication in the human genome mostly in the last phylostrata (10–17) [56], can slightly modify the pattern of the cancer/normal (or invasive/non-invasive) fold in these phylostrata (Figure 4A). Their effect is more pronounced in the comparison of polyploid and diploid cancer cells, which will be described later. For now, it is notable that a minor distortion of the ‘gradual atavism’ in the invasive/non-invasive cancer cells (the fold increase in 10–17 phylostrata compared with 7–9 phylostrata) cannot be explained by the higher expression of TF ZF-C2H2 (Figure 4C).

### 2.4. Polyploid Cancer Cells

**Figure 5:**
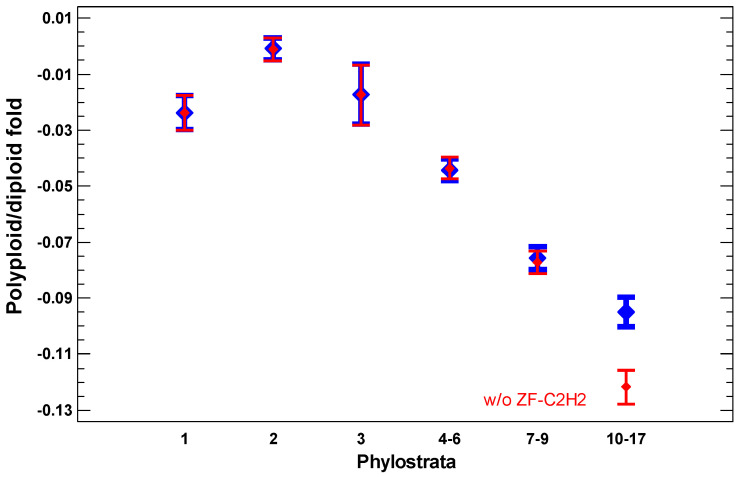
The evolutionary profile of gene expression fold in polyploid/diploid cancers (‘pancancer’, the data integrated over about 10,000 cancer samples). The data without ZF-C2H2 genes are shown in red. The strong activation of C2H2-ZF genes in polyploid cancer cells can be seen. Phylostrata: 1—cellular organisms (Prokaryota); 2—Eukaryota; 3—Opisthokonta; 4—Metazoa; 5—Eumetazoa; 6—Bilateria; 7—Chordata; 8—Vertebrata; 9—Euteleostomi; 10—Tetrapoda; 11—Amniota; 12—Mammalia; 13—Theria; 14—Eutheria; 15—Boreoeutheria; 16—Primates; 17—Hominidae.

The presence of polyploid cells in cancers is associated with a poorer prognosis [58,59,60,61,62,63]. The polyploid cancer cells show a further gradual enhancement of expression of more ancient genes as compared with diploid cancer cells (‘gradual atavism’) (Figure 5). The only difference with the cancer/normal and invasive/non-invasive folds is that genes from the second phylostratum show a higher fold than genes from the first phylostratum. This picture is in accordance with the interactome centrality measures and random walk modeling, which show a similar distortion among 1–2 phylostrata. This is probably because chromosome/chromatin maintenance (involving genes from the second phylostratum) is more important than metabolic activity (involving genes from the first phylostratum) for polyploid-diploid cancer cell transition compared with the basic cancer-normal cell transition. Because polyploidization presents the progression of cancer, the increase in the polyploid/diploid cancer fold supports the ‘serial atavism’ model [34].

Importantly, the ZF-C2H2 TF showed a significantly higher polyploid/diploid cancer fold than the other genes from 10–17 phylostrata (Figure 5). These TFs suppress mobile elements (MEs) by initiating their heterochromatinization [64,65]. The MEs are activated under stressful conditions because of chromatin opening and remodeling, and ZF-C2H2 TFs are upregulated to counteract ME activity [64,65]. Therefore, the upregulation of ZF-C2H2 TFs in polyploid cancer cells suggests an activation of MEs, which is in agreement with the ‘genome chaos’ theory.

### 2.5. Functional Analysis of Upregulated Genes in Cancer Cells

**Figure 6:**
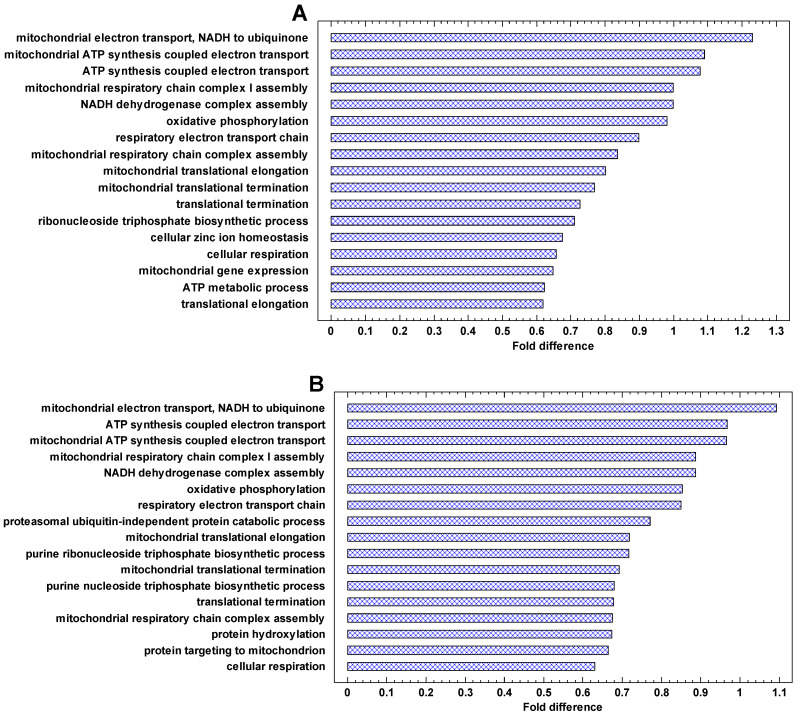
The most strongly enriched GO Biological Processes in the genes that are upregulated in cancers. Fold difference: the difference between the mean cancer/normal expression fold for a given process and the mean fold for all processes (log2). The processes with the highest folds (and having >20 genes) are shown: (**A**) breast cancer (*p* < 0.0001 at least); (**B**) melanoma (*p* < 0.0001 at least).

**Figure 7:**
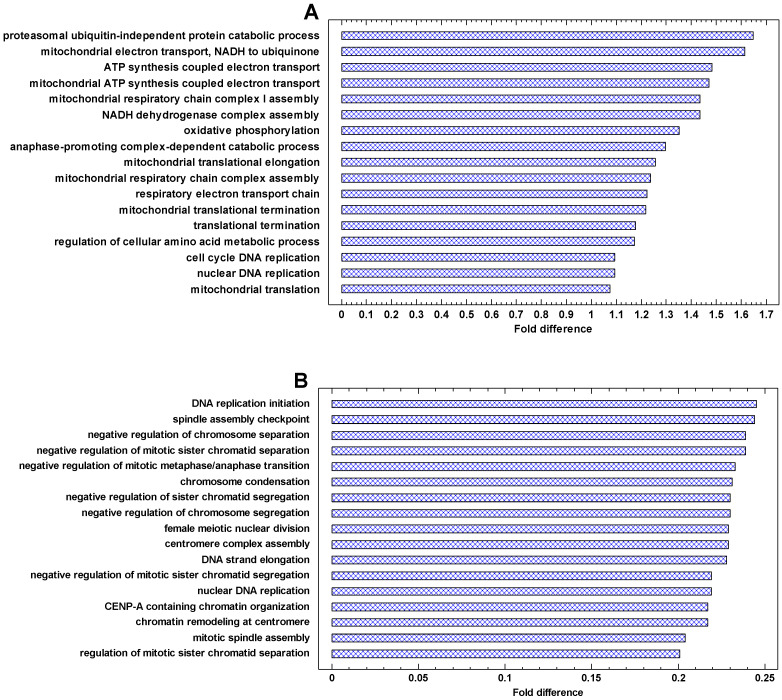
The most strongly enriched GO Biological Processes in the genes that are upregulated in the invasive and polyploid cancers. Fold difference: the difference between the mean cancer/normal expression fold for a given process and the mean fold for all processes (log2). The processes with the highest folds (and having >20 genes) are shown: (**A**) invasive/non-invasive fold (myeloma) (*p* < 0.0001 at least); (**B**) polyploid/diploid fold (‘pancancer’, the data integrated over about 10,000 cancer samples) (*p* < 0.0001 at least).

The genes that are most strongly upregulated in cancer cells are involved in energetics and translation (Figure 6 and Figure 7A). In the invasive vs. non-invasive cancer cells, DNA replication is added (Figure 7A). In polyploid vs. diploid cancer cells, the processes dealing with chromosomes and DNA replication are the most strongly upregulated processes (Figure 7B). This observation is in agreement with the phylostratic distribution of the cancer/normal fold in the UC region. This fold is higher in the first phylostratum (metabolism) for the cancer/normal and invasive/non-invasive comparisons (Figure 4), but it is higher in the second phylostrata for the polyploid/diploid comparison (Figure 5). This fact suggests that processes dealing with genetic information are more important in the polyploidization of cancer cells, whereas the metabolic boost is more prominent in the cancer-normal transformation. Notably, the ‘female meiotic nuclear division’ is among most strongly upregulated processes in polyploid/diploid cancer cells (Figure 7B). 

### 2.6. Functional Analysis of Downregulated Genes in Cancer Cells

**Figure 8:**
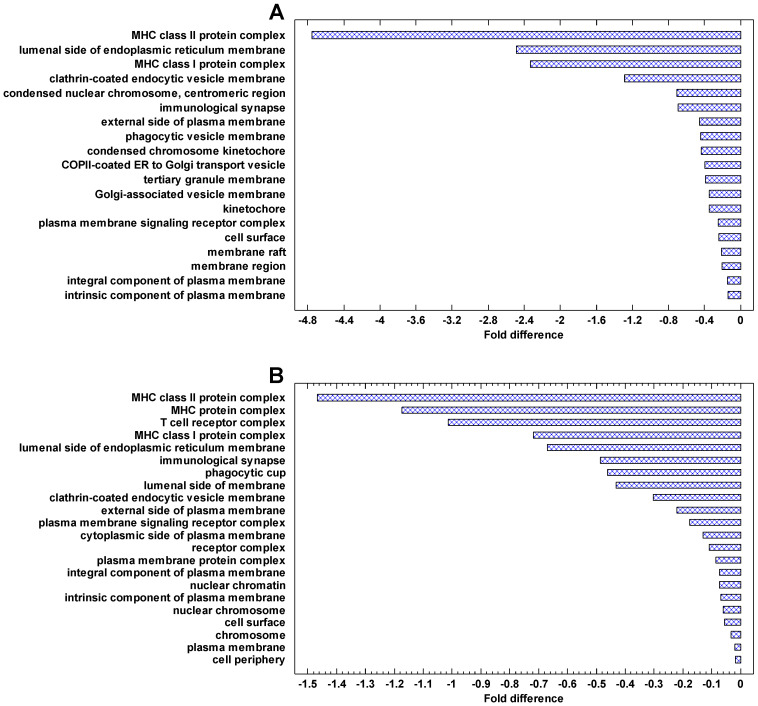
The most strongly enriched GO Cell Components in the genes that are downregulated in cancers. Fold difference: the difference between the mean cancer/normal expression fold for a given cell component and the mean fold for all cell components (log2). The components with the highest folds (and having >10 genes) are shown: (**A**) breast cancer (*p* < 0.0001 at least); (**B**) melanoma (*p* < 0.01 at least).

**Figure 9:**
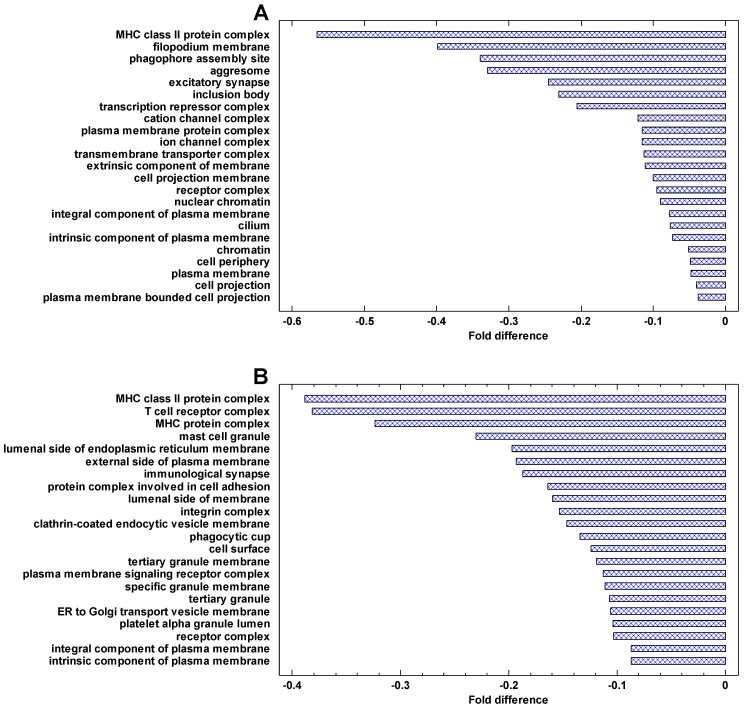
The most strongly enriched GO Cell Components in the genes that are downregulated in the invasive and polyploid cancers. Fold difference: the difference between the mean cancer/normal expression fold for a given cell component and the mean fold for all cell components (log2). The components with the highest folds (and having >10 genes) are shown: (**A**) invasive/non-invasive fold (myeloma) (*p* < 0.01 at least); (**B**) polyploid/diploid fold (‘pancancer’, i.e., the data integrated over about 10,000 cancer samples) (*p* < 0.0001 at least).

The genes, which are most strongly downregulated in cancer cells, are involved in the immune activity (especially the major histocompatibility complex, MHC), plasma membrane, phagocytic and Golgi-associated vesicles (Figure 8). The same can be seen in the genes downregulated in the invasive/non-invasive and polyploid/diploid cancer cells (Figure 9). These observations suggest that communication with the extracellular environment and compliance with immune surveillance (realized through the MHC) are suppressed in cancer cells and further suppressed in invasive and polyploid cancer cells. This observation is in agreement both with the TOFT and the SMT, because suppression of communication with the extracellular environment can be stipulated by the changes in this environment and/or realized via the genetic and epigenetic changes within the cell.

### 2.7. Evolutionary Origin of Cell Stress and Cell Cycle Genes

**Figure 10:**
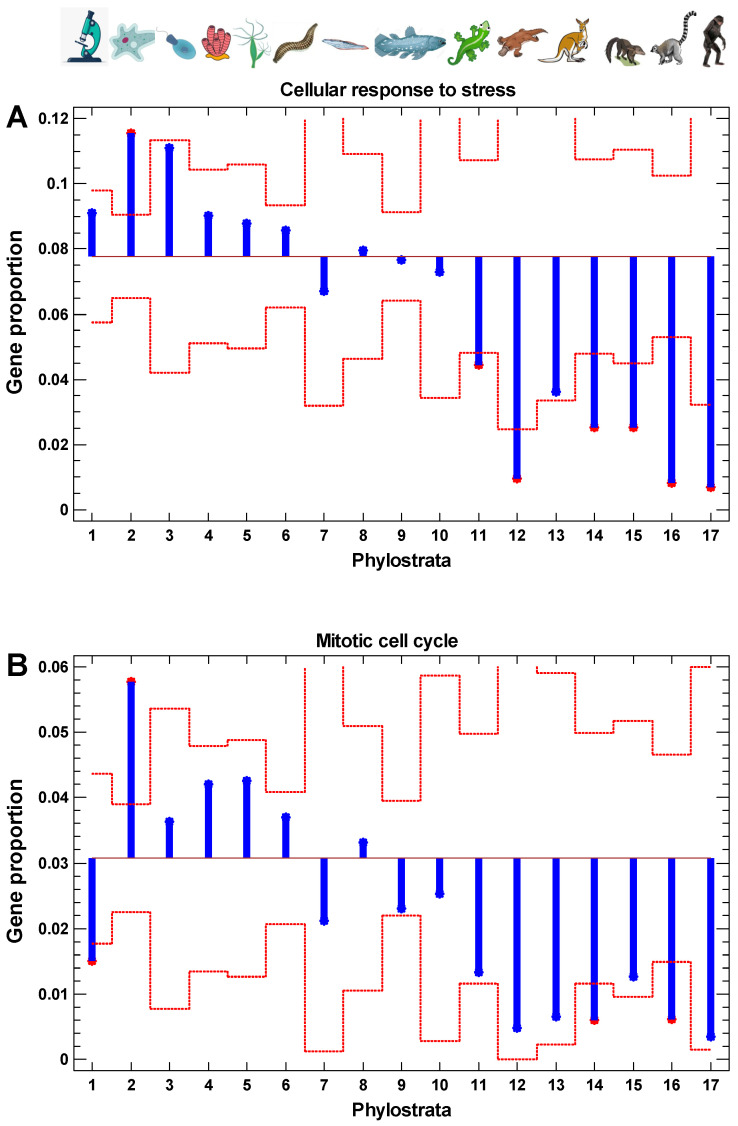
The evolutionary profile of genes belonging to GO Biological Processes: (**A**) ‘cellular response to stress’ (GO:0033554); (**B**) ‘mitotic cell cycle’ (GO:0000278). Red dotted lines show confidence intervals for individual phylostrata (*p* = 0.05). For all phylostrata taken as points, Spearman rank correlation between gene proportion and phylostrata: *r* = −0.96, *p* < 0.0001 (**A**); *r* = −0.82, *p* < 0.001 (**B**). Phylostrata: 1—cellular organisms (Prokaryota); 2—Eukaryota; 3—Opisthokonta; 4—Metazoa; 5—Eumetazoa; 6—Bilateria; 7—Chordata; 8—Vertebrata; 9—Euteleostomi; 10—Tetrapoda; 11—Amniota; 12—Mammalia; 13—Theria; 14—Eutheria; 15—Boreoeutheria; 16—Primates; 17—Hominidae. The pictures at the top show recent organisms corresponding to phyletic branching used for human gene dating.

The phylostratic distribution of genes involved in the cellular response to stress and the cell cycle reminds the distribution of interactome centrality, the results of random walk modeling along protein interaction trajectories, the levels of gene expression, and the cancer/normal, invasive/non-invasive, and polyploid/diploid folds (Figure 10). The only exception exists with the cell cycle genes of prokaryotic origin (first phylostratum), which suggests that informational processes dealing with epigenetic regulation (chromatin and chromosome maintenance and modification), appearing in the second phylostratum, are more important for cell cycle activity than metabolic pathways (appearing mostly in the first phylostratum). 

## 3. Discussion

### 3.1. General Model

The human interactome shows the gradual core-to-periphery evolutionary growth, which results in the higher protein interaction density and global interactome centrality in the UC center of cellular networks. The random-walk modeling demonstrates that the UC center serves as an attractor for random steps along protein interaction trajectories (even if started from the youngest proteins and normalized to gene number in each evolutionary stage). These observations suggest that random alterations in the interactome caused by genetic and epigenetic changes can result in the shift of protein interaction activity towards the UC center. The strength of the UC attractor gradually declines towards younger proteins. 

Similarly, gene expression is also higher in the UC center and gradually declines towards younger genes, indicating that the UC center is maintained in the more active state, compared with more recent periphery. In cancer cells, this effect is enhanced, and in invasive cancer cells, it is enhanced further. The most strongly upregulated processes in cancer cells include energetics and translation. In invasive vs. non-invasive cancer cells, DNA replication is added. In both comparisons (cancer vs. normal and invasive vs. non-invasive), the genes from the first phylostratum (Prokaryota) are the most strongly upregulated, which corresponds with the functional analysis revealing energetics and translation as the most strongly activated processes. This feature is in minor disagreement with the phylostratic pattern of gene expression, interaction density, interactome centrality, and random walk modeling in normal cells, where the genes from the second phylostratum (unicellular Eukaryota) show the highest values. This deviation indicates an important role of metabolic boost in cancer cells, whereas in normal cells, the informational processes (regulation of transcription and chromatin modification) are more prominent.

Collectively, these data suggest that the UC center of cellular networks, which already in normal cells is more active compared with the later network layers, further activates in cancer cells. Furthermore, the activation of gene expression shows not only the UC/MC contrast but also a gradual decline across the MC phylostrata towards more recent genes, which can be called the ‘gradual atavism’. This activation is enhanced in invasive and polyploid cancer cells. Because invasive and polyploid cancer cells present the progression of cancer, these observations support the ‘serial atavism’ model, which states that cancer onset and progression involve a series of atavistic reversals [34].

The UCA model does not contradict the SMT because it suggests that activation of UC center and relaxation of MC control can be caused by somatic mutations. However, the difference between the cell-centered UCA and the gene-centered SMT is that the critical genetic and epigenetic changes causing oncogenesis may belong to a broad gene spectrum, not necessarily limited to specific genes (oncogenes or oncosuppressors). For instance, the expression array analyses of different breast cancers showed that different gene sets were equally useful in predicting future clinical behavior but contained almost no genes in common [9,10]. Furthermore, both the increase and decrease in the same molecular actor can correlate positively with tumor malignancy [8]. 

The genes that are most strongly downregulated in cancer cells are involved in the immune activity (especially the major histocompatibility complex, MHC), plasma membrane, phagocytic and Golgi-associated vesicles. These cellular network modules are involved in communication with the extracellular environment and compliance with immune surveillance. Thus, conformity with the MC control, which counteracts the activity of the UC attractor in normal cells, is suppressed in cancer cells. These observations suggest an important role of extracellular effects in maintaining the normal cell state, which is in agreement with the ‘tissue organization field’ theory (TOFT) [8,45,46]. The TOFT states that cancer is a tissue-based disease. However, these observations do not contradict the SMT because suppression of communication with the extracellular environment can be realized via genetic and epigenetic alterations within the cell.

As the SMT cannot completely explain oncogenesis, the same can be said about the TOFT. Both molecular and biophysical components of the stroma can drive cell fate commitment in opposite directions, even in the presence of the same stimulus [8]. In other words, tissue effects also do not determine cell fate completely. Furthermore, TOFT states that proliferation is the default state for all cells [45,46], which is an overstatement because, in the culture (when there are no tissue constraints), cells can proliferate only in the presence of externally provided growth factors [45,46]. In addition, even with growth factors, the proliferation of adult organism cells is constrained by the Hayflick limit [53,54]. However, after the replacement of ‘default proliferation’ by the UC attractor causing the within-cell alterations, the TOFT can be reconciled with these observations. 

The genes involved in cellular stress response are mostly of UC origin. Therefore, the upregulation of these genes under stressful conditions can further activate the UC center, thus becoming a potential first step to oncogenesis. The prolonged intensive stress may fix this hyperactivation epigenetically. If stress occurs during the cell cycle, this effect may be even stronger because the cell cycle genes are also mostly of UC origin. In addition, stress increases the mutation rate due to both direct damage by stressful conditions (e.g., by reactive oxygen species) and ancient error-prone DNA repair, especially during the cell cycle when the genome occurs in a most vulnerable state, called ‘proliferation stress’ [66,67,68]. The relationship between the tissue proliferation activity and the probability of cancer was shown [69].

Being random alterations, mutations can shift the activity of cellular systems towards the UC attractor (according to the results of random walk modeling) and destroy mechanisms of MC control. When the MC control weakens because of genetic and epigenetic changes, the activity of cellular networks further shifts towards the UC center (manifested in the hyperactivation of UC genes in cancer cells), causing a loss of tissue-specific cell functions (dedifferentiation) and unleashing selfish cell behavior directed at survival by all means. 

**Figure 11:**
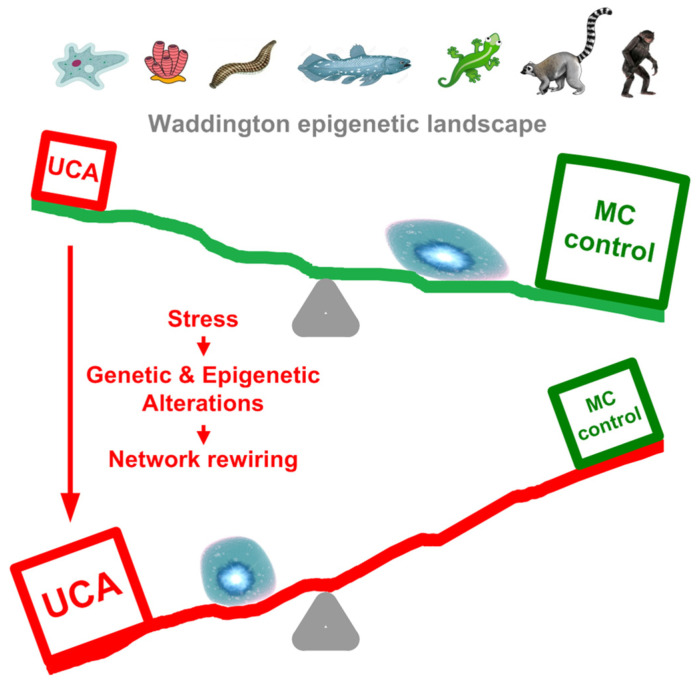
The Waddington epigenetic landscape of ontogenesis (reflected in cell differentiation) and its reversal by the activation of UC attractor as a result of genetic and epigenetic alterations caused by external and endogenous stress. The evolutionary stages at the top are visualized by recent representatives. (According to the biogenetic law, ontogenesis roughly recapitulates phylogenesis [57,72]).

Thus, there can be a synergism between the high gene expression in the UC center and the UC interactome attractor that is triggered by intensive cell stress, especially during cell proliferation. As a result, the Waddington epigenetic landscape of ontogenesis [70,71], which, in accordance with the biogenetic law, roughly recapitulates phylogenesis [57,72], can turn over. (The biogenetic law was validated on the cellular level [57].) In normal cells, this landscape is slanted towards cell differentiation, yet under stressful conditions it can be counteracted by the activity of UC attractor, causing landscape turnover and cell dedifferentiation (Figure 11). This turnover can occur in a series of steps, according to the ‘serial atavism’ model.

### 3.2. Cell Learning, Genetic Recombination, and Metastases

The hyperactivation of energetics in cancer cells is probably a reaction to prolonged intensive stress and the search for survival. This search can activate not only the ancient UC programs but also create novel programs via adaptation. Importantly, metabolic reprogramming in cancer cells is associated with the epigenetic remodeling via chromatin opening [73]. The search for novel pathways to survival is necessary because stressed cells found themselves under altered conditions of MC organisms, which were not previously met during both UC and MC life, and therefore require novel solutions. Recent studies showed that individual cells demonstrate exploratory learning for adaptation to novel conditions, which is not dependent on pre-existing pathways [50]. This learning is probably performed by epigenetic remodeling, which searches via trial and error for gene expression patterns allowing survival under novel stressful conditions. Thus, the yeast cells confronted with a severe challenge (media to which their biochemical networks were not adapted), while not dividing, continue to intensively metabolize and finally find an adapted network configuration and resume proliferation [74]. This adaptation requires a significant amount of energy; supposedly it is performed via non-genetic means because the adaptation rate is orders of magnitude higher than expected based on known mutation rates [74]. 

Notwithstanding their overall reversal to a UC-like state, selfish cancer cells can obtain at their disposal all genetic and epigenetic arsenals, accumulated during the MC evolution: the exploitation of the microenvironment, stimulating vascularization, immune response modulation, cell cooperation, etc. The cancer-associated fibroblasts, i.e., fibroblasts compelled to help cancer cells, are one of the examples [75,76]. Cell cooperation promotes many of the hallmarks of cancer via the secretion of diffusible factors affecting cancer cells or stromal cells in the tumor microenvironment [77]. The acquiring of the genetic and epigenetic arsenal, which was developed in MC evolution but not specific for a given cell type, is especially spectacular for metastatic cells invading alien environments. Many organ-specific adaptations of metastatic cells in the lung, bones, brain, and liver were identified [78]. For instance, most cancer cells from lung or breast cancer that infiltrate the brain will die, yet some of them acquire an ability to express brain-specific protective factors (plasminogen activator inhibitory serpins), allowing their survival [79]. In the liver, metastatic cells from colorectal cancer adapt their metabolic pathways to the hepatic environment [80]. Such adaptations require the activation of tissue-specific pathways that are not specific for progenitors of metastatic cells, which is probably achieved via network rewiring by epigenetic remodeling. The resistance of cancer cells to chemical treatment (not previously met both in UC and MC life) arises practically for all drugs that target specific molecules [81]. This resistance can arise by epigenetic mechanisms [26,27,29]. Similarly, the acquired epigenetic and transcriptional changes are critical drivers of metastasis [28]. The bivalent genes, which enable rapid switching between cellular programs, are probably involved in this epigenetic remodeling [61]. 

Genetic recombination caused by chromosome rearrangement may also have adaptive significance, which is suggested by the ‘genome chaos’ theory [18,30]. Genetic recombination may allow finding solutions for problems, which neoplastic cells encounter under novel stressful conditions. There may be an analogy with the alteration of sexual and asexual (clonal) generations in animal and plant populations. Sexual reproduction appears under worsening (stressing) conditions and allows finding adapted genomic variants using genome recombination [82,83]. Similarly, in cancer cells, the activation of the recombination-based adaptive search increases cell diversity, and some cells may acquire solutions to encountered problems. Notably, while 80% of cancer cells which invade the circulation system manage to survive and extravasate, only a very minor part (<0.02%) form macro-metastases [50].

Furthermore, even the normal cells contain a delay-action bomb—the selfish mobile elements (ME). They are suppressed by heterochromatinization initiated by C2H2 zinc fingers appearing in waves from the beginning of cellular life [56]. Most genes belonging to old waves were lost, leaving only small remnants [56]. These waves probably reflect the bursts of ME activity. The MEs are activated under stressful conditions because of chromatin opening and remodeling [64,65]. This is a general problem of the multilevel organization where the higher levels control the lower ones, which under stressful conditions can get out of control. Similarly, the MC organisms contain a delay-action bomb of potentially selfish cells. Metaphorically, one can consider oncogenesis as a ‘cell riot’ getting out of organismal control, which can be associated with a ‘genomic parasite riot’ getting out of cellular control, both arising because of stress [84]. The storm of activated ME enhances genome chaos and may participate in the search for genomic configurations adapted to novel stressful conditions. Propagation of MEs can alter gene expression patterns because they may insert in regulatory regions [64,65]. The activity of MEs leads to random genetic and epigenetic alterations, which further shift cellular network activity towards the UC attractor.

### 3.3. Polyploidization

The polyploid cancer cells show a further gradual enhancement of ancient gene expression, indicating that polyploidization presents the next stage in cancer progression. In polyploid vs. diploid cancer cells, the most strongly upregulated processes are DNA replication and chromosome processing. In agreement with this observation, genes from the second phylostratum show a higher upregulation than genes from the first phylostratum. In cancer vs. normal cells, the metabolic boost is stronger than the upregulation of processes dealing with genetic information. On the contrary, in polyploid vs. diploid cancer cells, the processes dealing with genetic information are activated stronger than metabolism. Notably, even in normal mammalian cells, polyploidization shifts gene expression towards more ancient genes [85]. 

Polyploidy results from the overall instability of stressed cancer cells [58,61,63,86,87]. This is probably because of the competition between the cell cycle and cellular stress response. The importance of this competition for polyploidization was reported even for functional stress [88,89]. Stress caused by diseases, which results in the formation and survival of polyploid cells, can be considered as an analog of environmental stress conferring an adaptive advantage to polyploid organisms [63]. 

Polyploidization makes cancer cells even worse because of chromosomal instability caused by difficulties with chromosome pairing and segregation [58,59,60,61,63]. Another recently recognized factor of gene expression changes in polyploid cells is the opening of chromatin owing to a decrease in surface/volume ratio, which relaxes chromatin architecture because of the loss of interactions of nuclear lamina with lamina-associated domains [90]. Chromatin opening may also cause cell dedifferentiation. Notably, the pluripotency signature (PluriNet) is upregulated in polyploid vs. diploid cancer cells, whereas the genes involved in regulation of multicellular organismal development (associated with cell differentiation) are downregulated [63].

Polyploid cancer cells show a general increase in adaptivity, which is reminiscent of the rapid growth, stress resistance, and the evolutionary plasticity of polyploid organisms [63]. Neoplastic cells also demonstrate higher adaptability for growth under stressful conditions because of the relaxation of cell cycle checkpoints, which can cause polyploidization. Thus, under the action of a chemical tumor promoter, human lymphocytes in primary culture continued DNA synthesis even when mitosis or cytokinesis were blocked by colchicine or cytochalasin, thereby forming polyploid cells [91]. The authors concluded that pretumor and tumor cells have more flexibility compared with normal cells, which stop growth when coming across any hindrance in their stringently programmed performance. Therefore, tumor cells can better adapt to varied conditions, and “such adaptability reflects the transition from cellular to organismal level of biological integrity (because, unlike a normal cell, tumor cell can be considered as a unicellular organism)” [91]. 

Importantly, rodent cells are more prone to malignant transformation than human cells [19,20,21]. This is probably because of the relaxed cell cycle control in these rodents, where natural selection favors the speed of development and reproduction at the expense of the reliability of cellular processes [22,23]. This feature is reminiscent of the action of a tumor promoter. For instance, the mitotic spindle assembly checkpoint is relaxed in the mice and hamsters compared with humans, which is caused by the evolutionarily conserved MAD1 gene mutation [92]. The transfection of the human MAD1 into the mouse and hamster cells corrected the relaxed checkpoint to a more stringent form [92]. The spindle assembly checkpoint fidelity is positively correlated with the body mass of adult mammal species [93]. In a similar vein, cardiac interstitial tetraploid cells can escape replicative senescence in murine models but not large mammals (humans and swine) [94]. These observations suggest that relaxed cell cycle control, caused either by tumor promoters or evolutionary trade-offs, can be associated with the easiness of malignant transformation and polyploidization.

Among the most strongly upregulated GO processes in polyploid vs. diploid cancer cells is the ‘female meiotic nuclear division’. This observation confirms previous reports on the activation of meiotic genes in polyploid cancer cells [95,96,97,98]. This fact can be a sign of genetic recombination, which is reminiscent of sexual generations arising in apomictic (clonal) animal and plant populations under stressful conditions and may be inherited from the evolutionary past. This assumption is in agreement with the ‘genome chaos’ model suggesting the adaptive nature of chromosomal instability in cancer cells [18,30]. In addition, polyploid cancer cells show a higher expression of C2H2 zinc finger transcription factors, compared with diploid cancer cells. The main function of C2H2-ZF is the counteraction of mobile elements (ME) [64,65]. This observation suggests the activation of ME in polyploid cancer cells, which can enhance genome chaos.

### 3.4. Conclusions

On the grounds of the meta-analyses performed here, we propose a unicellular attractor (UCA) model integrating the essential features of the ‘atavistic reversal’, ‘cancer attractor’, ‘somatic mutation’ (SMT), ‘genome chaos’, and ‘tissue organization field’ (TOFT) theories put forward earlier. The ‘atavistic reversal’ is taken as a keystone. We propose a possible mechanism of this reversal, its refinement called the ‘gradual atavism’, and evidence for the ‘serial atavism’ model. The UCA model suggests that the UC attractor arises owing to the gradual core-to-periphery evolutionary growth of cellular networks resulting in the higher protein interaction density and global interactome centrality in the UC center. Even in the normal cells, the ancient genes are more actively expressed. Random walk modeling along protein interaction trajectories suggests that random alterations in cellular networks caused by genetic and epigenetic changes can result in further shifts of network activity towards the UC center. These changes can be caused and accelerated by cellular stress, which additionally activates UC genes, especially during cell proliferation, because genes involved in cellular stress response and cell cycle are mostly of UC origin. Genetic and epigenetic changes can also disrupt tissue control over individual cells because cancer cells demonstrate the downregulation of genes involved in communication with the extracellular environment (especially in immune surveillance). The UCA model does not contradict the SMT because it suggests that the activation of UC attractor and the relaxation of MC control can be caused by somatic mutations (as well as epigenetic alterations). However, the difference between the cell-centered UCA and the gene-centered SMT is that critical genetic and epigenetic changes can belong to a broad gene spectrum, not necessarily limited to specific genes (oncogenes or oncosuppressors). The activation of gene expression is gradually declined towards more recent genes, which was called the ‘gradual atavism’. In invasive and polyploid cancer cells (both presenting cancer progression), gene hyperactivation is further shifted towards the UC center, also in agreement with the ‘serial atavism’ model. Collectively, these events may unleash selfish cell behavior aimed at survival at all means. While the selfish behavior of neoplastic cells is probably triggered by the activation of ancient UC programs, it can be realized not only by these programs but also by MC programs non-specific for a given cell type and by new programs created via network rewiring afforded by epigenetic remodeling (cell learning) and genetic recombination (‘genome chaos’). In the case of genetic recombination, only a minor part of novel genome configurations can be adaptive, which results in the clonal evolution of cancer cells. 

### 3.5. Possible Limitation and Future Prospective

Albeit the single-cell transcriptome datasets studied here being limited to three cancer types, they are very different cancers, including invasive and non-invasive forms. Furthermore, for polyploid/diploid cancers, the ‘pancancer’ data were used, which were integrated over about 10,000 samples of very different cancer types [60]. The main results were consistent for all datasets. The phylostratigraphy of the human interactome, the evolutionary course of its centrality measures, and the random walk modeling across protein interaction trajectories are of a general nature, not limited to a cancer type. The same is relevant to the phylostratigraphic analysis of the genes beloning to the cell cycle and cellular stress response.

We hope that the UC attractor model could facilitate an understanding of oncogenesis and promote the diagnostics and development of therapeutic strategies. The ratio of expression of unicellular genes to multicellular ones can be used in the diagnostics for cancer grading and prognosis. The genes and proteins of unicellular origin should probably be targeted predominantly so as to overcome the activity of the UC attractor. For instance, certain unicellular-specific drugs can be applied for this purpose [36,99]. The systemic nature of oncogenic alterations suggests the necessity in multi-target strategies against the unicellular genes whose expression is enhanced most drastically in cancer cells. The extracellular systemic alterations suppose an important role of immunotherapy (in combination with other treatments), which now achieved certain advancement [100,101,102,103]. The extracellular matrix “normalization” can also be proposed as a potential strategy for anti-malignant treatment [104]. As for regenerative medicine, healthy regeneration could involve an ontogenetic reversal to a younger organism’s state (which, according to the biogenetic law, corresponds to earlier multicellular stages) without a phylogenetic reversal to a unicellular cell state [57].

## 4. Materials and Methods

### 4.1. Interactome and Random Walk Modeling

The human pairwise protein interactions were acquired from the STRING database [55]. We selected the interactions with a top-half confidence (>0.5), which is slightly higher than default confidence used by the STRING server (>0.4). The number of direct (one-step) interactions and the measures of global centrality (betweenness and closeness) for each protein were determined using Cytoscape [105] (version 3.9.1).

**Figure 12:**
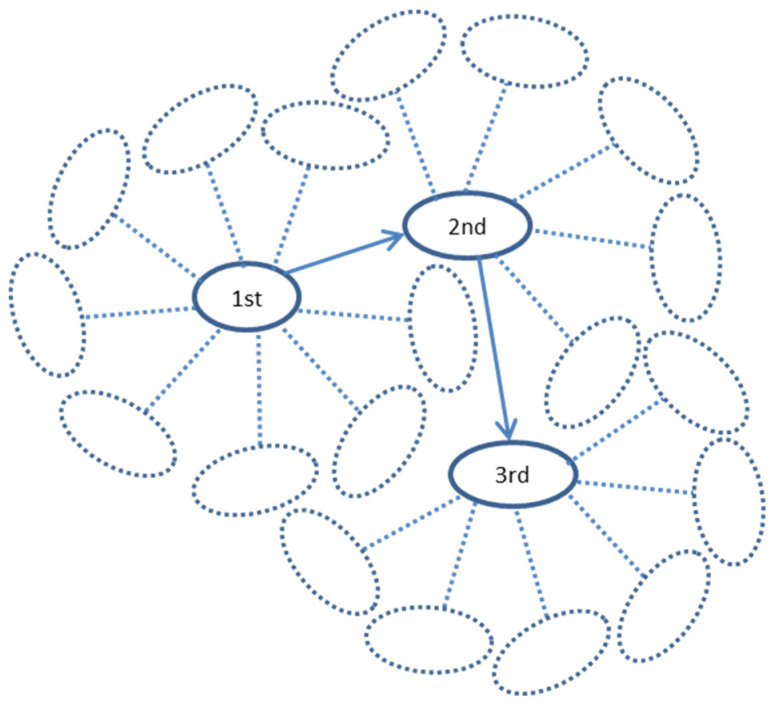
The diagram showing the random walk modeling along the protein interaction trajectories in the human interactome. A walk started from one of the youngest proteins (belonging to 17th phylostratum), taken randomly. This was the 1st protein. From all its interactants, one was chosen randomly (2nd protein), and the next step started already from this protein, again to a randomly chosen next interactant (3rd protein), and so on. The reverses to 1st and other previous proteins were allowed. The series of walks of a different length (from 5 to 10,000 steps) were tested.

The random walk modeling along the protein interaction trajectories in the human interactome was performed as follows (Figure 12). A walk started from one of the youngest proteins (belonging to 17th phylostratum), taken randomly. This was the 1st protein. From all its interactants, one was chosen randomly (2nd protein), and the next step started already from this protein, again to a randomly chosen next interactant (3rd protein), and so on. The reverses to 1st and other previous proteins were allowed. The series of walks of a different length (from 5 to 10,000 steps) were tested. To ensure statistical significance, there were 10,000 repeats of each random walk, each repeat starting randomly from one of the proteins in 17th phylostratum. The number of repeats, which ended in each phylostratum, was normalized to the number of genes belonging to this phylostratum. We assume that these random walks can simulate random alterations in the interactome caused by mutations in the encoding gene or disturbances in gene expression or protein configuration. It was shown previously that alterations in protein interactions are associated with changes in gene expression [57].

### 4.2. Cancer and Normal Cell Transcriptomes

The human cancer and normal single-cell transcriptomes were acquired from the Gene Expression Omnibus [106]. The datasets were the ‘breast cancer’ GSE75688 [107], ‘melanoma’ GSE72056 [108], and ‘myeloma’ GSE106218 [109]. These datasets were chosen because they are single-cell transcriptomes (allowing analyses of homogenous cells), each containing the transcript levels both for cancer and normal cells obtained by the same sequencing method in the same laboratory. The transcript levels (called in the text ‘expression’ for brevity) were normalized separately for each dataset using the ‘limma’ software implemented in the R package (with the ‘quantile’ normalization method). The limma seems a most universal approach for disparate datasets because it can treat both natural (counts) and real numbers [110]. The limma makes log2-transformation. Then the log-transformed values were averaged for each gene across all either cancer or normal cells (separately in each dataset). The cancer/normal folds for each gene were calculated by subtraction of the mean of cancer cells from the mean of normal cells. The mean log-transformed transcript levels for cancer and normal cells or cancer/normal folds of the genes belonging to a tested gene group (e.g., genes belonging to a phylostratum or GO category) were averaged for this gene group in each dataset. The data on genes, which are differentially expressed in polyploid vs. diploid cancer cells, were acquired from [60]. They contained only the polyploid/diploid folds for cancers. The ‘pancancer’ data (i.e., integrated over about 10,000 samples of different cancer types) were taken.

### 4.3. Phylostratigraphy and ZF-C2H2 Genes

The evolutionary stratification of human genes (phylostratigraphy, or gene dating) was acquired from [56], where the problems of different gene dating methods were discussed (shallow vs. deep). Here, we used the shallow phylostratigraphy, which is based on the strict gene orthology obtained using the best reciprocal hits with the accurate Smith–Waterman algorithm. (In contrast, the deep phylostratigraphy includes in-paralogous genes, thus providing dating of whole gene families.) The list of ZF-C2H2 genes was acquired from the InterPro database [111]. The genes encoding for proteins containing the zinc finger C2H2 superfamily (IPR036236) domain were selected.

### 4.4. Enriched Gene Modules

The functional enrichment analysis (presented in Figure 6, Figure 7, Figure 8 and Figure 9) was performed by the contrast test as described previously [22,23,88]. In this test, the mean parameter of genes belonging to each Gene Ontology (GO) category is compared with the mean parameter of total gene set. For each GO category, we collected all its subcategories using GO-directed acyclic graphs (DAG), and a gene was regarded as belonging to a given category if it was mapped to any of its subcategories. This is necessary because many genes are mapped in the GO database only to their specific categories and not to a general category. As an example, only one gene is mapped to the protein modification process (GO:0036211) directly, whereas 2500+ genes can be mapped to this process using the GO DAG because protein modifiers are distributed across specific protein modification processes. 

The evaluation of statistical significance was conducted by the Monte Carlo method as described previously [22,23,88]. For estimation of two-tailed significance of the contrast between the mean cancer/normal (or polyploid/diploid) fold of a process/pathway and the corresponding mean value of a total gene set, we conducted for each GO category 20,000 random samplings without replacement from the total gene dataset (with complete replacement after each sampling). The size of random samples was equal to the number of genes in a tested process/pathway. This procedure is analogous to random gene permutation (shuffling) when each GO category randomly acquires genes from the total dataset. The means of random samples were compared with the mean of genes belonging to a tested GO category. Depending on how frequently the random sample mean is higher (or lower) than the mean of a tested GO category, the significance was calculated. This method is preferable to parametric or non-parametric tests because the normal distribution that is required for parametric tests is usually absent, whereas non-parametric tests can lose a considerable amount of information. The random-sampling test is distribution-independent (because random sampling follows the dataset distribution) and retains all information. The correction for multiple comparisons was performed according to [112]. This procedure gives the q-value (false discovery rate), which can be considered as the *p*-value corrected for multiple comparisons. 

## Data Availability

The data underlying this article are available in the article.
